# Factors influencing the informal caregiving needs among the older adult population in Jiangmen City, China: a cross-sectional study

**DOI:** 10.3389/fpubh.2025.1620957

**Published:** 2025-09-04

**Authors:** Yue Zhou, Huailei Zhang, Shan He, Siqi Huang, Xin Zhang, Fang Fu, Guanghua Zhang

**Affiliations:** ^1^School of Nursing, Guangdong Jiangmen Chinese Medicine College, Jiangmen, China; ^2^Wuyi Hospital of Traditional Chinese Medicine, Jiangmen, China; ^3^School of Nursing, Huizhou Health Sciences Polytechnic, Huizhou, China

**Keywords:** informal care, training needs, current situation investigation, caregiving, cross-sectional study

## Abstract

**Objective:**

Although China has a vast population, less attention has been paid to the status and training needs of informal caregivers. This study aimed to assess the training needs of the informal caregivers in Jiangmen city, located in Guangdong Province in southern China.

**Methods:**

Three communities within a district of Jiangmen City were selected, and primary caregivers of older adults were invited to complete a self-developed training needs questionnaire. A total of 250 questionnaires were distributed, and 237 questionnaires were finally collected. Of the collected questionnaires, 214 (90.4%) were deemed valid.

**Results:**

Informal caregivers reported a substantial caregiving burden. About 78.5% of informal caregivers had not received formal caregiving training, and over 91.1% expressed a need for such training. In terms of training content, the dimension of older adults care knowledge received the highest scores, followed by health care techniques and daily living care skills, while rehabilitation care techniques scored the lowest. The mean item score was 4.17 ± 0.88. Multi-stepwise regression analysis indicated that training needs of informal caregivers were significantly associated with age, education, employment status, and relationship to the care recipient.

**Conclusion:**

There is a strong demand for informal caregiver training in Jiangmen, Guangdong. Training needs were significantly associated with caregivers’ age, education, employment status, and relationship to the care recipient.

## Introduction

Population aging is a great challenge globally due to increasing life expectancy and decreased fertility rate, especially in mainland China with a vast population (1, 2). A trend similarly is observed in many developed countries, including the USA, UK, France, and Germany (3). On this basis, home-based informal caregiving, community-based residential care, and institutionalized care have been advocated and become increasingly significant modes of care provision. According to Article 49 of the Constitution of the People’s Republic of China, *“Parents have the duty to rear and educate their minor children, and children who have come of age have the duty to support and assist their parents”* (4). This constitutional provision reflects the traditional Confucian value of filial piety, which remains deeply embedded in Chinese culture and social norms. On this basis, most of the older adults care is given by their family members.

Informal caregiving refers to the unpaid care and assistance provided primarily by family members, relatives, or close friends to older adults with functional limitations. As a type of care usually provided by family members, it assists the older adults with functional limitations in their daily lives with no paid compensation (5). Informal caregiving for the older adults is often a long-term commitment, involving not only basic daily support but also more complex tasks such as rehabilitation training and the management of chronic diseases (6). The scope and intensity of caregiving tasks vary widely, depending on the number of caregiving hours, specific care needs, and the level of support available. Compared with the nursing caregivers, informal caregivers, typically family members without formal training, are more likely to experience significant physical and psychological stress (7). The lack of professional knowledge and caregiving skills often contributes to a gradual decline in care quality, negatively affecting the health and well-being of older adults over time.

Studies in the USA and the UK have demonstrated that untrained informal caregivers are at increased risk of caregiver burden, depression, and reduced care quality (8). Evidence from randomized controlled trials and systematic reviews shows that targeted caregiver training programs can significantly reduce caregiving burden, enhance coping skills, and improve outcomes for both caregivers and care recipients (9). Despite a vast population in China, limited attention has been paid to the status and training needs of informal caregiving in the Chinese population. Previous research found that greater caregiving load was significantly associated with higher caregiver burden, with social support intensity playing a key moderating role (10). However, while the impact of caregiving burden has been acknowledged, relatively little is known about the specific skills and support informal caregivers require to improve care quality and reduce stress. Accordingly, this study sought to identify the training needs of informal caregivers in Jiangmen City, China, with a focus on the types of caregiving skills required, preferred training formats, and factors associated with those needs.

## Materials and methods

### Subjects

This questionnaire-based survey was conducted via an online platform,^1^ and participants were recruited from three residential communities in three communities from a district of Jiangmen City, located in Guangdong Province in southern China. Jiangmen is part of the economically developed Pearl River Delta region and has a rapidly aging population due to both urbanization and increasing life expectancy. The selected communities represent a mix of urban and suburban areas, with varying levels of access to healthcare and eldercare resources, making them suitable for assessing the training needs of informal caregivers. The inclusion criteria were as follows: (a) individuals who self-identified as the primary caregivers for disabled older adults, defined as persons with significant physical or cognitive impairments, or diagnosed with Alzheimer’s disease, who required assistance with basic life support, disease caring and rehabilitation; (b) caregivers who provided such care without receiving any financial compensation; (c) possessed sufficient cognitive and communication ability to understand the study’s purpose and procedures and to provide informed consent; and (d) voluntarily participate in this survey. The individuals with paid compensation, with bias in the understanding of this study, or not willing to participate in the survey were excluded from this study. Caregiving roles and care recipient conditions were verified through screening questions at the beginning of the questionnaire. Participants were asked to indicate the relationship to the care recipients, the types of care tasks they performed (e.g., feeding, bathing, medication administration), and the specific health conditions of the older adults person they cared for. The ethical approval was waived by the board of the ethics committee of our college.

### Questionnaire designing and validation

To ensure the scientific validity and relevance of the questionnaire content, a two-round Delphi method was employed. A total of 20 experts in geriatric care were invited to participate, including professionals from hospitals, long-term care institutions, and academic settings located in Guangzhou, Wuhan, and Beijing (Supplementary File 1). Among them, 13 experts held senior or associate senior professional titles, 12 held a master’s degree or higher, and all had more than 5 years of experience in older adults care or related fields. In the first round, experts were asked to rate the relevance and clarity of each item on a 5-point Likert scale and provide qualitative suggestions for revision. Feedback was analyzed quantitatively and qualitatively. Items with a mean score below 4.0 or a coefficient of variation (CV) greater than 0.25 were subject to revision or removal. In the second round, the revised questionnaire was redistributed, and experts re-evaluated the items. Kendall’s coefficient of concordance (W) was calculated to assess the level of consensus, with *W* > 0.70 indicating strong agreement. After the second round, consensus was reached on all items, and the final version of the questionnaire was established based on expert feedback and a small-scale pilot test.

The questionnaire consisted of three parts: (i) The questionnaire for collecting the demographic information, including sex, age, marriage, employment, education, health condition, relationship with the older adults in care, and the assistance involved in the care. (ii) Informal caregiving information, including hours spent in caregiving, average hours spent in caregiving per day, source of caregiving knowledge. (iii) Training-need assessment scale for informal caregivers. It comprised 18 items, organized into four logically related dimensions established through the two-round Delphi process: (a) older adults caregiving knowledge (five items), covering psychological and physiological characteristics of older adults, common chronic diseases, medication guidance, and nutritional/dietary needs; (b) life-care technology (five items), focusing on daily living support skills such as feeding, bathing, dressing, toileting, and safe mobility assistance; (c) health caring technology (five items) including disease monitoring, first aid for emergencies (e.g., falls, choking, injuries), infection prevention, and basic nursing procedures; and (d) rehabilitation caring (two items) involving guidance on functional training and mobility rehabilitation for older adults. The number of items per dimension in the training needs scale reflects the relative importance and demand for different types of elder care knowledge and skills, resulting in the observed differences across dimensions. A scale of 1–5 was designated for each item, with 1 score demonstrating not necessary and 5 score demonstrating very necessary. Caregivers selected the appropriate score based on their own conditions. A high score indicated a high training need for the informal caregiving. The scale had been tested for reliability and validity, showing an acceptable Cronbach’s Alpha coefficient.

### Methodology

After obtaining the consent of the community committee, the questionnaires were distributed to the survey respondents who met the inclusion criteria from April 2023 to October 2023. The investigators of this research team were all qualified and received professional training before distributing the questionnaire in filling in the remarks during the survey, distributing and collecting the questionnaires, as well as timely answers to questions raised by the survey respondents. A total of 250 questionnaires were distributed, and 237 questionnaires were finally collected. Among the collected questionnaires, 214 (90.4%) questionnaires were effective. A questionnaire was considered valid if it met the following criteria: all essential items were completed without missing data; the responses were internally consistent (e.g., no logical contradictions); and the respondent met the inclusion criteria. We applied multi-stepwise regression to identify the key factors influencing the training needs. This approach allows us to systematically evaluate multiple potential predictors while controlling for confounding variables, and to select the most significant variables that contribute to variations in caregivers’ training needs.

### Statistical analysis

After removing the invalid questionnaires, the data from the eligible questionnaires were entered into the Excel. All the data were analyzed using the SPSS 26.0 software. Continuous variables normally distributed were expressed as mean ± standard deviation (SD). Descriptive statistical analysis was used to analyze the scores of each dimension of the demographic information, caregiving data, and training need scale. Student’s *t*-test and variance analysis were used to analyze the needs of respondents with different characteristics. Variables with a *p* value of less than 0.05 in the univariate regression analysis was adopted into the multiple stepwise regression, to identify the risk factors for the training need of informal caregiving. A *p* value of less than 0.05 was considered to be statistical significance.

## Results

### Basic demographics of the informal caregivers

In total, 214 subjects (female: 153; male: 61) accomplished the questionnaire. The majority of informal caregivers (62.1%) were aged 40 years or older. Merely 22.4% of the caregivers obtained a bachelor’s degree or more. About 56.6% of the caregivers showed a son/daughter-in-law or daughter/son-in-law relationship with the older adults care recipients. In addition, 19.6% of the caregivers showed a history of chronic disease (Table 1).

**Table 1:** General information of informal caregivers.

Variables	Number (percentage)
Gender	
Male	61 (28.5%)
Female	153 (71.5%)
Age	
<30 years	50 (23.4%)
30–40 years	31 (14.5%)
40–50 years	91 (42.5%)
>50 years	42 (19.6%)
Marriage	
Married	146 (68.2%)
Not-married	61 (28.5%)
Divorced and/or widowed	7 (3.3%)
Employment	
Full-time employment	95 (44.4%)
Part-time employment	34 (15.9%)
No job	53 (24.8%)
Retired	16 (7.5%)
Farmer	16 (7.5%)
Education	
Preliminary school	21 (9.8%)
Middle school	44 (20.6%)
High school	42 (19.6%)
College	59 (27.6%)
Bachelor’s degree or more	48 (22.4%)
Relationship	
Spouse	12 (5.6%)
Son/daughter-in-law	74 (34.6%)
Daughter/son-in-law	47 (22.0%)
Other relations	60 (28.0%)
Social relationship (e.g., friends or neighbors)	21 (9.8%)
Family assistance during the informal caregiving	
Yes	109 (50.9%)
No	105 (49.1%)
Monthly income	
≤2,000 CNY	57 (26.6%)
2,000–4,000 CNY	51 (23.8%)
4,000–6,000 CNY	55 (25.7%)
>6,000 CNY	51 (23.8%)
Health status of the caregivers	
Good	140 (65.4%)
Moderate	66 (30.8%)
Poor	8 (3.7%)
Chronic diseases	
Yes	42 (19.6%)
No	172 (80.4%)

### Training needs of informal caregivers

Table 2 summarized the caregiving characteristics of the informal caregivers. About 42.5% had been providing care for up to 12 months, followed by those caring for 3 months or less (38.8%), 3–6 months (14.7%), and 6–12 months (4.7%). In terms of average daily caregiving time, 43.9% reported less than 2 h per day, followed by 2–6 h (37.9%), 6–12 h (12.1%), and more than 12 h (6.1%). The majority of caregivers (78.5%) had not received any professional training prior to providing informal care. Among all respondents, 61.2% expressed a perceived need for professional caregiving training. Among those who had received training, most had only been trained in basic life support (23.9%), with the primary sources of training being experienced friends or family members.

**Table 2:** Caregiving situation of informal caregivers.

Variables	Number (frequency)
Total caregiving time	
<3 months	83 (38.8%)
3–6 months	30 (14.0%)
6–12 months	10 (4.7%)
>12 months	91 (42.5%)
Caregiving time per day	
<2 h	94 (43.9%)
2–6 h	81 (37.9%)
6–12 h	26 (12.1%)
>12 h	13 (6.1%)
Received professional training on the caregiving	
Yes	46 (21.5%)
No	168 (78.5%)
Necessity for the training on caregiving	
Extremely	83 (38.8%)
Yes	112 (52.3%)
No	19 (8.9%)
Received training for the caregiving	
Care positioning, quality and ability recognition	17 (12.0%)
Physical and ability assessment	28 (19.7%)
Basic life care	34 (23.9%)
Medication	22 (15.5%)
Care for functionally impaired older adults people	13 (9.2%)
Emergency rescue	13 (9.2%)
Dementia care	6 (4.2%)
Palliative care	9 (6.3%)
Tools for obtaining the caregiving knowledge	
Friends and relatives with care experience	139 (26.1%)
Professional nursing	89 (16.7%)
Online resource and television program	105 (19.7%)
Books	70(13.1%)
Education from elders	95 (17.8%)
Care-related lectures and forums	35 (6.6%)

### Score of training needs for informal caregivers in various dimensions

Table 3 presented the training needs score of informal caregivers. Among the four dimensions of the training needs assessment scale, older adults caregiving knowledge received the highest mean score (4.17 ± 0.88), followed by health care technology (4.14 ± 0.82), life-care technology (4.04 ± 0.95), and rehabilitation care (3.92 ± 1.05). Within the training needs scale, two items showed a mean score of less than 4, including functional impairment (i.e., speech, movement, swallowing) and user guides to the commonly utilized facilities (e.g., wheelchairs and walkers). The top needs for the informal caregiving training were the necessities for the caring of chronic diseases including diabetes mellitus (DM), hypertension and chronic obstructive pulmonary disease (COPD) (Table 4).

**Table 3:** Training needs scores of informal caregivers in various dimensions.

Variables	Number of question	Score
Older adults care knowledge	5	4.17 ± 0.88
Life care knowledge	5	4.04 ± 0.95
Health care knowledge	6	4.14 ± 0.82
Rehabilitation care knowledge	2	3.92 ± 1.05
Total	18	4.10 ± 0.77

**Table 4:** Score on training needs items among informal caregivers.

Dimension	Entry	Score
Older adults care knowledge	Guidance on knowledge related to the physiological and anatomical characteristics of the older adults	4.00 ± 1.07
	Nursing guidance for common psychological and mental problems	4.19 ± 0.97
	Guidance on common chronic diseases in the older adults such as DM, hypertension, and COPD	4.29 ± 0.94
	Diet and nutrition guidance	4.24 ± 0.95
	Daily life and TCM healthcare knowledge guidance	4.04 ± 1.04
Life care knowledge	General dietary care techniques for the eating and drinking	4.08 ± 1.04
	Guidelines on providing oral, hair, and skin care, as well as morning and evening hygiene support	4.04 ± 1.04
	Guidance on excretion care techniques for constipation and urinary incontinence	4.00 ± 1.01
	Sleep care	4.00 ± 1.00
	Technical guidance on safety and transfer care	4.07 ± 1.02
Health care knowledge	Guidance on drug-use safety	4.20 ± 0.91
	Guidance on measuring vital signs including temperature, respiration, pulse and blood pressure	4.14 ± 0.88
	Special dietary care guidance	4.13 ± 0.96
	Guidance for the caring of the older adults with Alzheimer disease	4.05 ± 1.04
	First aid guidance for the older adults on falling, choking, injuries, and fractures	4.27 ± 0.85
	Palliative care guidance	4.09 ± 0.96
Rehabilitation care knowledge	Rehabilitation training and care guidance for older adults people with functional impairment in language, movement and swallowing	3.94 ± 1.09
	Guidance on the use of common assistive devices for the older adults such as wheelchairs and walkers	3.90 ± 1.04

COPD, chronic obstructive pulmonary disease; DM, diabetes mellitus; TCM, traditional Chinese medicine.

### Multiple stepwise regression analysis

With the needs score as the dependent variable, we adopted the variables with statistical significance (*p* < 0.05) in the univariate analysis into the multiple stepwise regression analysis. Based on univariate analysis (Table 5), informal caregivers with different age, working status, educational level and relationship with the older adults under care showed different training needs.

**Table 5:** Univariate analysis for the training needs of the informal caregivers.

Variables	Number	Score	*t*-value	*P*-value
Gender			−1.749	0.084
Male	61	3.93 ± 0.98		
Female	153	4.16 ± 0.66		
Age			13.620	<0.001
<30 years	50	3.67 ± 0.83		
30–40 years	31	3.76 ± 1.07		
40–50 years	91	4.32 ± 0.47		
>50 years	42	4.38 ± 0.66		
Marriage			0.779	0.460
Married	146	4.13 ± 0.72		
Not-married	61	4.07 ± 0.82		
Divorced and/or widowed	7	3.77 ± 1.28		
Employment			5.423	<0.001
Full-time employment	95	3.84 ± 0.93		
Part-time employment	34	4.24 ± 0.70		
No job	53	4.33 ± 0.42		
Retired	16	4.44 ± 0.51		
Farmer	16	4.21 ± 0.49		
Education			6.914	<0.001
Preliminary school	21	4.58 ± 0.94		
Middle school	44	4.42 ± 0.81		
High school	42	3.99 ± 0.56		
College	59	4.01 ± 0.60		
Bachelor’s degree or more	48	3.79 ± 0.82		
Relationship			4.242	0.003
Spouse	12	4.42 ± 0.72		
Sons or daughters-in-law	74	4.17 ± 0.55		
Daughters or sons-in-law	47	4.33 ± 0.51		
Other relations	60	3.89 ± 0.89		
Social relationship (e.g., friends or neighbors)	21	3.74 ± 1.22		
Family assistance during the informal caregiving			−0.399	0.690
Yes	109	4.08 ± 0.85		
No	105	4.12 ± 0.68		
Monthly income			1.828	0.143
≤2,000 CNY	57	4.26 ± 0.62		
2,000–4,000 CNY	51	3.99 ± 0.78		
4,000–6,000 CNY	55	3.97 ± 0.77		
>6,000 CNY	51	4.16 ± 0.88		
Health status of the caregivers			0.914	0.403
Good	140	4.06 ± 0.81		
Moderate	66	4.13 ± 0.70		
Poor	8	4.42 ± 0.41		
Chronic diseases			−0.141	0.888
Yes	42	4.08 ± 0.84		
No	172	4.10 ± 0.75		

The multiple stepwise regression analysis results are shown in Table 6. Compared with caregivers aged <30 years, those aged 40–50 years (*B* = 0.51, 95% CI: 0.25 ~ 0.76, *p* < 0.001) and 50 years or more (*B* = 0.43, 95% CI: 0.14 ~ 0.73, *p* = 0.004) had significantly higher training needs. Relative to full-time workers, unemployed (*B* = 0.71, 95% CI: 0.46 ~ 0.95, *p* < 0.001) and retired caregivers (*B* = 0.46, 95% CI: 0.10 ~ 0.82, *p* = 0.01) reported higher needs. Higher education was linked to lower needs, with college and bachelor’s degree or above scoring lower than primary education (*B* = –0.42, 95% CI:–0.75 ~ −0.09, *p* = 0.012). Compared with sons- or daughters-in-law and daughters or sons-in-law, other relatives (*B* = –0.41, 95%CI: −0.65 ~ −0.17, *p* < 0.001) and non-relatives (*B* = –0.57, 95%CI: −0.88 ~ −0.25, *p* < 0.001) had significantly lower scores.

**Table 6:** Multiple stepwise regression analysis results with demand score as the dependent variable.

Variables	Unstandardized coefficients	95% CI	*t*-value	*P*-value
Aged less than 30	Reference			
Aged 30–40 years	0.02	−0.29 to 0.34	0.14	0.89
Aged 40–50 years	0.51	0.25–0.76	3.97	<0.001
Aged 50 or more	0.43	0.14–0.73	2.90	0.004
Full-time occupation	Reference			
Part-time occupation	0.18	−0.09 to 0.45	1.33	0.18
No job	0.71	0.46–0.95	5.72	<0.001
Retired	0.46	0.10–0.82	2.50	0.01
Farmer	0.20	−0.16 to 0.56	1.08	0.28
Preliminary school	Reference			
Middle school, high school	−0.31	−0.63–0.02	−1.88	0.06
College, bachelor’s degree or more	−0.42	−0.75 to −0.09	−2.54	0.012
Sons or daughters-in-law, daughters or sons-in-law	Reference			
Spouse	−0.04	−0.44 to 0.36	−0.21	0.83
Social relationship (e.g., friends or neighbors)	−0.41	−0.65 to −0.17	−3.36	<0.001
Other relations	−0.57	−0.88 to −0.251	−3.54	<0.001

## Discussion

Caring the older adults presents a significant challenge for families, particularly in China, where population aging is accelerating. In some developed countries, family caregiving has been incorporated into the long-term care insurance system (11, 12), however, in most of Asian countries, adult children are expected to assume primary responsibility for supporting and caring for their aging parents, due to cultural norms of filial piety and legislative requirements (13). This reliance on informal caregiving has led to considerable burdens for family members. Thus, attention should be paid to these individuals in order to alleviate their stress and improve the quality of life (QoL) for both caregivers and care recipients. This study was designed to investigate the current landscape of informal caregiving in Jiangmen City and to identify the factors associated with caregiving needs, based on findings from a cross-sectional survey.

Informal caregiving has been associated with reduced quality of life, poorer overall well-being, increased risk of severe depression, and substantial financial strain (14). Despite these challenges, most informal caregivers have limited access to professional training and receive no financial compensation for their efforts (15, 16). In the present study, 47.2% of informal caregivers have provided care for more than 6 months, and 56.1% offered care for more than 2 h per day, and 18.2% reported caregiving durations exceeding 6 h daily. Additionally, over half of the respondents were not engaged in full-time employment while providing care, reflecting the significant time commitment and economic burden associated with informal caregiving. These results were consistent with the previous studies (14). Notably, 78.5% of informal caregivers had not received any form of professional training, and over 90% expressed a need for such training. These results implied the urgent and unmet demand for structured training programs to support informal caregivers in delivering safe and effective care. In our survey, some caregivers had received only basic life-support training, with a lack of professional guidance. Informal caregivers primarily acquired caregiving knowledge through informal channels, such as advice from friends or relatives with caregiving experience, online resources and television programs (17). Very few participants had access to structured training delivered by formal institutions or professional caregivers. Consistent with the previous studies (18, 19), our data showed that the caregiving responsibilities were predominantly undertaken by middle-aged, unemployed women. These findings underscore the critical need for accessible, standardized, and professionally led training programs to better equip informal caregivers in China.

Our study showed a substantial demand for caregiving training among informal caregivers, with an overall score of 4.17 ± 0.88. Among the four dimensions, knowledge related to older adults care received the highest score, encompassing psychological and physiological guidance, information on common chronic diseases, and dietary and nutritional advice. This elevated need may reflect the high prevalence of chronic conditions among older adults (20), for which caregivers require more specialized knowledge, including medication management and disease-specific dietary recommendations. The score for rehabilitation caring was the lowest among these items. This may be attributed to the perception that rehabilitation should be conducted by trained professionals, such as physicians or institutional nurses, rather than informal caregivers. Among the 18 items assessed across the four dimensions, the top three priorities identified were: guidance on managing common chronic diseases in older adults (e.g., hypertension, DM, and COPD); first aid procedures (e.g., for falling, choking, injury and fracture); and diet and nutrition guidance. These findings are in line with previous studies, which have consistently shown that informal caregivers express the most urgent need for knowledge regarding chronic disease management (21), first aid (22), and older adults nutrition (23).

Caregivers of different age groups showed different needs for the informal caregiving training (19). Our findings indicate that older caregivers, particularly those aged 40–50 years and above, reported higher training needs, which may reflect their more intensive caregiving roles, longer daily care hours, and potentially lower adaptability to new caregiving knowledge acquisition channels compared with younger caregivers. In contrast, younger caregivers may rely more on self-learning via digital resources, reducing their perceived need for formal training. This may be attributed to their younger age and generally higher educational attainment, which enables them to access caregiving knowledge independently through the internet, books, and other self-directed channels (24). Education level was also a key determinant, with lower educational attainment linked to higher demand for training. This aligns with the notion that caregivers with limited educational backgrounds may face greater challenges in understanding disease management, medication use, and rehabilitation techniques without structured guidance. This finding aligns with previous studies showing that lower educational attainment is associated with a higher perceived caregiving burden (25, 26). These findings suggest the importance of adopting stratified and tailored training strategies. For older caregivers with lower levels of education, easily accessible formats such as popular science videos, animated content, and other visually engaging materials may facilitate understanding and knowledge retention. In contrast, for more educated caregivers, expert-led seminars or in-depth lectures on geriatric conditions may offer a more appropriate and effective training modality.

Significant differences were reported in the training needs of informal caregivers with different working conditions (27). Employment status further influenced training demand: unemployed and retired caregivers expressed stronger needs in this study, likely due to their greater availability for caregiving and reliance on it as a primary daily activity. Balancing employment responsibilities with caregiving duties can constrain their involvement in direct care, thereby reducing their perceived need for formal training (28). This finding was consistent with the study by Rachiel, which showed that full-time employment was associated with reduced hours spent on informal caregiving as early as the 1980s (29). Therefore, for informal caregivers with full-time employment, training program should offer greater flexibility in terms of timing, delivery methods, and formats. This approach would enable them to access essential caregiving knowledge efficiently, without imposing additional time and energy burdens, thereby improving the quality of care they provide. In our study, significant differences were observed in the training needs and dimensions among informal caregivers with varying relationships to the older adults. Among them, compared with sons or daughters-in-law, and daughters or sons-in-law, those shared a social relationship or other relations with the older adults reported significantly lower training needs. This may reflect the closer familial or blood ties between these caregivers and the care recipients, which could foster a stronger intrinsic motivation to enhance their caregiving skills and provide higher-quality care.

There are inevitably some limitations in this study. First, this is a retrospective study, which could not eliminate the possibility of selection bias. Second, the sample size is not large, and in the future, we will carry out a study with a large sample size. Third, the study did not capture detailed information regarding the specific training content desired by respondents, nor did it account for the potential use of alternative or supplementary caregiving arrangements. Forth, training dimensions were not grouped as logically and systematically as might be ideal, which may affect the clarity and readability of the framework. Last, the generalizability of these findings may be limited due to the unique social, cultural, and economic characteristics of Jiangmen. Differences in caregiving practices, family structures, and available resources across regions could influence the applicability of the results to other settings. Due to many direct relatives living abroad, most caregivers were other relatives or friends, potentially affecting generalizability. Therefore, caution should be exercised when extrapolating these findings beyond Jiangmen, as training needs and caregiving dynamics may vary in different contexts.

In summary, there is a great need for informal caregiver training in Jiangmen city, Guangdong, China. More than half of the informal caregivers had not received formal training in older adults care and demonstrated a strong demand for such training. Multi-stepwise regression analysis indicated that training needs were significantly associated with caregivers’ age, education level, employment status, and relationship to the care recipient.

## Data Availability

The original contributions presented in the study are included in the article/Supplementary material, further inquiries can be directed to the corresponding author.

## Footnotes

^1^ https://www.wjx.cn/
