# Supplementary material for: Factors influencing the informal caregiving needs among the older adult population in Jiangmen City, China: a cross-sectional study
Source: Front Public Health. 2025 Sep 4;13:1620957. doi: 10.3389/fpubh.2025.1620957 (PMC12443532; doi:10.3389/fpubh.2025.1620957)
Supplement: Supplementary file 1 [file Table_1.docx]

**Questionnaire on the training needs of informal caregivers of caregiving-dependent elderly people**

Thank you very much for your active participation in this questionnaire survey. In order to better understand the training needs of informal family caregivers for professional care knowledge and to improve the care level of informal caregivers, we are now investigating the training needs of informal caregivers who care for dependent elderly people. Your choice plays an important role in the standardization and development of informal care!

I hope you will fill in the form truthfully, and we will keep your information strictly confidential. The first part of the questionnaire is basic information, the second part is the current situation and attitude of care, and the third part is your need for training.

In order to save time and ensure efficiency, please fill in the items one by one in order, and do not leave any items blank. After filling in, please check that there are no missing contents and submit the questionnaire. Finally, thank you very much for your support for this questionnaire! I wish you good health and good work!

**Part I: General Information of Informal Caregivers**

1. Gender

| ○ Male |
| --- |
| ○ Female |

2. Age

_________________________________

3. Marriage

| ○ Married |
| --- |
| ○ Unmarried |
| ○Divorced or widowed |

4. Your occupation

| ○ full time job |
| --- |
| ○ part time job |
| ○ no job |
| ○ retired |
| ○ farmer |

5. Education

| ○ Preliminary school |
| --- |
| ○ Middle school |
| ○ High school |
| ○ College |
| ○ Bachelor’s degree or more |

6. Relationship to the caregiving-dependent people

| ○ Spouse |
| --- |
| ○ Son/daughter-in-law |
| ○ Daughter/son-in-law |
| ○ Other relations |
| ○ Social relationship (e.g. friends or neighbors) |

7. Family assistance during the informal caregiving

| ○ yes |
| --- |
| ○ No |

8. Monthly income of the informal caregiver

| ○ ≤2000元 ≤2000 CNY |
| --- |
| ○ 2000-4000元2000-4000 CNY |
| ○ 4000-6000元4000-6000 CNY |
| ○ >6000元>6000 CNY |

9. The healthy status of the caregivers

| ○ Good |
| --- |
| ○ moderate |
| ○ poor |

10. Chronic diseases such as diabetes and hypertension

| ○ Yes |
| --- |
| ○ no |

**Part II: Caregiving information for informal caregivers**

11. How many elderly people do you need to care for?

_________________________________

12. How long has the elderly needed care?

| ○ <3 months |
| --- |
| ○ 3-6 months |
| ○ 6-12 months |
| ○ >12 months |

13. The time that the elderly must have someone to take care of them every day

| ○ <2 h |
| --- |
| ○ 2-6h |
| ○ 6-12h |
| ○ >12h |

14. Before providing care for an elderly person who is dependent on care, have you received professional training in care knowledge?

| ○Yes, please fill in question 15 |
| --- |
| ○no |

15. What kind of care knowledge training have you received? [Multiple choice] *

| □ Care orientation and quality and ability awareness |
| --- |
| □ Physical and ability assessment of the elderly |
| □ Basic life care |
| □ Medication care |
| □ Care for the functionally impaired elderly |
| □ Emergency First Aid |
| □ Dementia Care |
| □ Hospice Care |
| □ Others _________________ |

16. Do you think it is necessary to receive professional care training?

| ○ Very much |
| --- |
| ○ yes |
| ○ no |

17. What is the source of your current care knowledge? [Multiple choice]

| □ Friends and relatives with care experiences |
| --- |
| □ Hospital professional nursing staff |
| □ network and TV |
| □ books |
| □ from the elders |
| □ Lectures and forums |

**Part III: Questionnaire on Training Needs for Informal Caregivers Caring for Dependent Elderly People (Please read the following questions and select the appropriate option based on the care training you would like to receive)**

18. Guidance on knowledge related to the physiological and anatomical characteristics of the elderly

○Very necessary

○Necessary

○No problem

○Not very necessary

○ Not necessary

19. Nursing guidance for common psychological and mental problems of the elderly

○Very necessary

○Necessary

○No problem

○Not very necessary

○ Not necessary

20. Guidance on common chronic diseases in the elderly (such as diabetes, hypertension, chronic obstructive pulmonary disease)

○Very necessary

○Necessary

○No problem

○Not very necessary

○ Not necessary

21. Diet and nutrition guidance for the elderly

○Very necessary

○Necessary

○No problem

○Not very necessary

○ Not necessary

22. General dietary care techniques for the elderly (eating and drinking)

○Very necessary

○Necessary

○No problem

○Not very necessary

○ Not necessary

23. Special dietary care guidance for the elderly

○Very necessary

○Necessary

○No problem

○Not very necessary

○ Not necessary

24. Technical guidance on oral, hair, skin, morning and evening cleaning and care for the elderly

○Very necessary

○Necessary

○No problem

○Not very necessary

○ Not necessary

25. Guidance on excretion care techniques for the elderly (constipation, urinary incontinence)

○Very necessary

○Necessary

○No problem

○Not very necessary

○ Not necessary

26. Sleep care technology for the elderly

○Very necessary

○Necessary

○No problem

○Not very necessary

○ Not necessary

27. Technical guidance on safety and transfer care for the elderly

○Very necessary

○Necessary

○No problem

○Not very necessary

○ Not necessary

28. Guidance on safe medication care for the elderly

○Very necessary

○Necessary

○No problem

○Not very necessary

○ Not necessary

29. Guidance on measuring vital signs (temperature, respiration, pulse, blood pressure) for the elderly

○Very necessary

○Necessary

○No problem

○Not very necessary

○ Not necessary

30. Rehabilitation training and care guidance for elderly people with functional impairment (language, movement, swallowing)

○Very necessary

○Necessary

○No problem

○Not very necessary

○ Not necessary

31. Care guidance for elderly people with dementia (senile dementia)

○Very necessary

○Necessary

○No problem

○Not very necessary

○ Not necessary

32. Emergency first aid guidance for the elderly (falls, choking, injuries, fractures)

○Very necessary

○Necessary

○No problem

○Not very necessary

○ Not necessary

33. Guidance on the use of common assistive devices for the elderly (such as wheelchairs, walkers)

○Very necessary

○Necessary

○No problem

○Not very necessary

○ Not necessary

34. Daily life and TCM health care knowledge guidance for the elderly

○Very necessary

○Necessary

○No problem

○Not very necessary

○ Not necessary

35. Palliative care guidance for the elderly

○Very necessary

○Necessary

○No problem

○Not very necessary

○ Not necessary
